# Mechanosensing and the Hippo Pathway in Microglia: A Potential Link to Alzheimer’s Disease Pathogenesis?

**DOI:** 10.3390/cells10113144

**Published:** 2021-11-12

**Authors:** Lucrezia Bruno, Simge Karagil, Almas Mahmood, Ahmed Elbediwy, Michael Stolinski, Francesca E. Mackenzie

**Affiliations:** 1 Department of Biomolecular Sciences, Kingston University London, Kingston-upon-Thames KT1 2EE, UK; k1463249@kingston.ac.uk (L.B.); k1931747@kingston.ac.uk (S.K.); m.stolinski@kingston.ac.uk (M.S.); 2 Department of Chemistry and Pharmaceutical Sciences, Kingston University London, Kingston-upon-Thames KT1 2EE, UK; k1515530@kingston.ac.uk

**Keywords:** Hippo, YAP, MST1, mechanotransduction, mechanosensing, microglia, Alzheimer’s disease, neurodegeneration, immunometabolism, neuroinflammation

## Abstract

The activation of microglia, the inflammatory cells of the central nervous system (CNS), has been linked to the pathogenesis of Alzheimer’s disease and other neurodegenerative diseases. How microglia sense the changing brain environment, in order to respond appropriately, is still being elucidated. Microglia are able to sense and respond to the mechanical properties of their microenvironment, and the physical and molecular pathways underlying this mechanosensing/mechanotransduction in microglia have recently been investigated. The Hippo pathway functions through mechanosensing and subsequent protein kinase cascades, and is critical for neuronal development and many other cellular processes. In this review, we examine evidence for the potential involvement of Hippo pathway components specifically in microglia in the pathogenesis of Alzheimer’s disease. We suggest that the Hippo pathway is worth investigating as a mechanosensing pathway in microglia, and could be one potential therapeutic target pathway for preventing microglial-induced neurodegeneration in AD.

## 1. Introduction

The role of inflammation in the pathogenesis of chronic diseases, including neurodegenerative disease, is an area of intense current research. Neuroinflammation is observed in multiple neurological diseases [1,2,3]. Alzheimer’s disease (AD) is the most common neurodegenerative disease, estimated to account for 60–70% of all cases of dementia worldwide [4]. Pathologically, AD is characterised by the presence of extracellular amyloid-beta (Aβ) protein aggregates, named plaques, as well as intracellular neurofibrillary tangles (NFTs) of hyperphosphorylated tau protein, in central nervous system (CNS) tissue, and widespread CNS neuronal cell death and brain tissue atrophy [4,5]. Both genetic and environmental factors are thought to contribute to AD development. Gene identification in familial or early-onset AD forms has discovered a number of genes involved, for example, in amyloid and associated protein processing that contribute to AD pathogenesis [5], and many more risk loci have been identified. According to the prevailing amyloid hypothesis, the deposition of amyloid-β (Aβ) in the brain is the determining event in AD, although evidence shows that this hypothesis alone is not sufficient to explain many aspects of AD pathogenesis [4] and is thus considered in combination with the tau-hyperphosphorylation theory of AD development. Tau protein is expressed in neurons and provides neuronal stability by stabilising the neuronal microtubule network [5], but tau hyperphosphorylation can be responsible for abnormal tau folding, decreasing its binding ability to microtubules and thus leading to microtubule degradation and tau protein self-aggregation [6]. The deposition of NFTs, formed by the aggregation of tau proteins, is therefore also a hallmark of AD pathogenesis [7]. Many recent findings have implicated microglia, the immune cells of the mammalian central nervous system (CNS), in the initiation and pathogenesis of Alzheimer’s disease and other neurological diseases featuring neuroinflammation, but the molecular mechanisms underlying this are not yet well understood. The Hippo pathway is a well-studied, conserved signalling pathway important in a variety of cellular processes, including mechanosensing. In this review, we examine evidence of a potential mechanotransductory role of the Hippo pathway in microglia and then consider what is known about the role of this pathway in microglia in the pathogenesis of AD.

## 2. Microglia and Their Role in the Pathogenesis of Alzheimer’s Disease

Microglia are macrophage-like cells resident in the mammalian brain and CNS [8]. In contrast to macrophages, they remain only within the CNS and have slow self-renewal [9]. Microglia constitute 0.5–16.6% of total cell population in the human brain [10] and about 5–20% of the total glial cell population in the rodent brain [11]. Since their discovery, different theories were developed about their origin. Pio del Rio-Hortega firstly introduced the hypothesis of a microglial mesodermal origin [12,13]. A widely supported theory suggests that microglial precursors (embryonic macrophages) originate from the yolk sac mesoderm during embryogenesis [12]; however, another hypothesis places microglial origin on the differentiation of microglyocytes from blood monocytes [14,15], with the latter theory originating from del Rio-Hortega, who suggested blood monocytes invade the CNS in prenatal stages [15].

**Figure 1:**
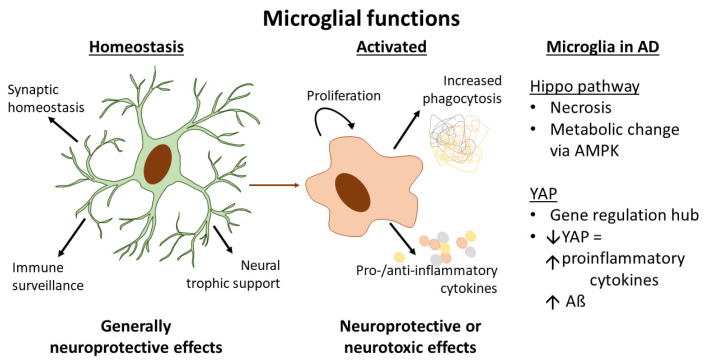
Functions associated with microglia in the homeostatic and activated states, including roles currently known and ascribed to microglial Hippo pathway components/YAP in Alzheimer’s disease (detailed in Section 4). During homeostasis, microglia perform functions in the CNS to support neurons and to surveil for pathogens or other debris. Once activated by a number of possible stimuli, microglia change gene expression and function to increase their phagocytic ability, and to resolve infection/damage by releasing pro- and/or anti-inflammatory cytokines. These activated functions may result in resolution of infection/damage and thus neuroprotection or, for example, in neurodegenerative diseases, result in continued inflammation and subsequent neurotoxic effects. Current evidence suggests that the Hippo pathway and YAP have important roles in microglia in the pathogenesis of AD. AD, Alzheimer’s disease; AMPK, AMP-activated kinase; YAP, Yes-associated protein; Aβ, amyloid beta.

Being a specialised type of immunological glia and being recognised as ‘protectors’ of the central nervous system, research interest in microglia has grown significantly over recent years. The CNS microenvironment affects microglial phenotype, allowing them to sustain their role in regulating and maintaining brain homeostasis, thereby providing a suitable microenvironment for the survival and function of neural cells [16,17]. When in a homeostatic state, microglia adopt a ramified form [18] and detect pathogens and cellular debris by extending their processes [19] (Figure 1). However, when the brain microenvironment is compromised, due to altered homeostasis or damage [20], microglia migrate to affected regions, proliferate, adopt an amoeboid shape, and change their phenotype to an ‘activated’ state to increase both phagocytosis of cellular debris and expression of inflammatory mediators [21] (Figure 1). Eventually, activated microglia cells experience apoptosis by an activation-induced cell death (AICD) process [22,23].

Microglia are activated by any type of pathologic insult to CNS tissue, including infection or injury [13]. Upon activation, microglia can release pro-inflammatory (e.g., TNF-α, IL-1β, IL-6, IL-8, or anti-inflammatory (e.g., TGF-β, IL-4, IL-10, IL-13) cytokines, leading to either neuroprotective or neurotoxic effects [24,25] (Figure 1). Therefore, depending on their activation, microglia can promote neuronal survival or neuronal degeneration [10,26]. For this and other reasons, microglia are suggested to be involved in the pathogenesis of neurodegenerative diseases [7].

Given the increased level of many inflammatory markers detected in AD patients (see [27] for a meta-analysis), neuroinflammation and microglia are therefore implicated with a critical role in AD development and progression [4,28,29]. It is unclear whether microglia-related inflammation occurs before or subsequent to the hyperphosphorylation of tau protein and Aβ deposition, but some studies have suggested that microglia-mediated inflammation precedes tau protein dysfunction. For example, Yoshiyama et al. [7] detected microglial activation in the tauopathy mouse model PS19 at 3–4 months old, when NFTs were still not detected. Furthermore, microglial inflammation was detected prior to Aβ plaque deposition in the AD 5xFAD mouse model [10]. Although it is not well characterised, microglia may also support the healthy clearance of amyloid accumulation and/or induce an inflammatory response in reaction to Aβ or tau protein [30].

Therefore, although the timing and function of microglial activation in AD pathogenesis is unclear, it is likely that they are related, which is also supported by evidence that AD risk genes are expressed in microglia. These include Apolipoprotein E (ApoE) and Triggering Receptor Expressed On Myeloid Cells 2 (TREM2) [31].

## 3. Mechanosensation in Microglia

Mechanosensing and mechanotransduction (the sensing of and response to physical or mechanical stimuli or forces in a cell’s microenvironment, by the cell) is critical for many biological processes, including cell growth, differentiation, cytoskeletal rearrangement, and cellular death. A number of cellular receptors (which can be described as ‘mechanosensors’) and pathways that respond to physical/mechanical stimuli have now been identified, including for responses to pressure, stretch, shear stress, substrate or matrix ridigity or ‘stiffness’, substrate topology, and other physical factors. Microglia are highly responsive to their environment, and change shape, migrate, and change function regularly to perform their roles in resolving inflammation, trophic support of other cells, synaptic homeostasis and other roles. To do this, microglia need to be able to respond to both molecular and physical environmental cues. The study of mechanosensing and mechanotransduction in microglia is a relatively new field of research, but a number of significant recent findings described below have begun to address the physical and molecular processes occurring.

### 3.1. Mechanobiology of the Microglial Microenvironment in the Brain during Development and in Alzheimer’s Disease

The brain and CNS are composed of relatively soft tissue, but they are reported to show spatial and temporal variations in their tissue rigidity (‘softness’ or ‘stiffness’) through development, aging, and in pathologies; the mechanisms are not well defined, but may be due to changes in cellular composition (e.g., increasing white matter or functional connectivity), extracellular matrix, vasculature, or brain water content (reviewed in [32,33]). For example, healthy cortex and corpus callosum show tissue stiffness compound modulus values of 4.83 and 32.15 kPa, respectively, for strain rates at 1 Hz, in rats [34] although similar studies report varying measurements. In the brain, the extracellular matrix (ECM) is a major determining factor of tissue stiffness and is made up of hyaloronic acid, chondroitin sulphate proteoglycans (CSPGs), tenasin-R, elastin, and laminin [33]. In recent years, many neural cell types have been shown to behave and function differently across variations in neural tissue stiffness (e.g., [35,36]) proving that, as was previously known with cancer cells, neuronal and glial cells can be highly mechanosensitive; these variations and cell responses are hypothesised or in some cases have been shown to cause appropriate developmental, aging or injury responses.

During human aging, brain ECM alters in composition [37]; this also occurs in the AD brain, where CSPGs are upregulated around amyloid plaques, abnormal collagen IV and fibronectin deposition occurs, and there is a loss of perineuronal nets (protective ECM structures) around neurons (see 33 for an excellent review of additional biomechanic changes in the AD brain). In addition, AD amyloid plaques are brittle and stiff: amyloid fibrils have high mechanical stiffness at 3.3 ± 0.4 GPa, as measured by Young’s modulus, making them more stiff than the surrounding brain tissue, and comparable in stiffness to spider silk [38]; and phosphorylated tau, as is present in NFTs in AD, is stiffer than non-phosphorylated tau [39]. However, conversely, exposure to Aβ reduces neuronal membrane stiffness in primary hippocampal neurons [40]; and on the macroscale, overall brain stiffness actually tends to decrease in AD (e.g., [41]), possibly due to widespread neuronal cell loss. Despite these findings, our understanding of the biomechanic changes in the AD brain is still lacking much detail [33].

In the case of microglia, these cells can sense mechanical properties of their substrates [32] and show migration towards stiffer substances [42], a process named durotaxis [43]. For example, during retinal development, microglia migrate through a stiffness gradient, and subsequently change their shape from ramified to bipolar-rod-like, a process which helps establish the vascular architecture of the developing retina [44]. In the context of microglia, soft PDMS-based elastic substrates (which vary between 1 and 0.6 kPa) have been reported to increase in vitro microglial proliferation and anti-inflammatory characteristics [45], whereas stiffer polyacrylamide/poly-d-lysine-based substrates (10–30 kPa) increase microglial pro-inflammatory characteristics [46]. It has therefore been suggested that brain tissue stiffness changes may be associated with the development of neurodegenerative pathologies; additionally, it has been suggested that microglia may act as mechanosensing cells in the pathogenesis of AD, or indeed other neurodegenerative diseases with neuroinflammatory components. For example, microglia may sense ECM- or amyloid-based stiffness in pre-AD brains (e.g., [47]), may migrate towards this stiffer material through durotaxis, and then respond either in healthy (e.g., anti-inflammatory) or detrimental (e.g., pro-inflammatory) ways, which affect the development or progression of neurodegeneration in AD [32].

### 3.2. Molecular Mechanisms of Mechanotransduction in Microglia

In contrast to the mechanical mechanisms of microglia, the molecular aspects of general mechanotransduction in this cell type have not been well elucidated.

Microglia express several mechanosensitive transient receptor potential (TRP) ion channels, including TRPV1, TRPV2, TRPV4, TRPM2, TRPM4, TRPM7, and TRPC3. These channels function in microglial activation, inflammation, clearing cellular debris, trophic support, and pain, particularly through modulating intracellular calcium homeostasis (see [48] for an excellent review). For example, approximately 60% of microglia express TRPV1 channels [49], which appear to partially mediate the cellular response to pressure in retinal microglia [50]. Additionally, recent studies have shown that TRPV4 channels mediate temperature-dependent [51] and stretch/osmolality-dependent [52] movement of retinal microglia. TRPV4 also regulates the macrophage inflammatory response to environmental stiffness [53]. It will be interesting to identify if cortical microglial TRPV1 and TRVP4 channels function in similar ways. While some TRP channels in microglia appear to function in a neuroprotective role (e.g., TRPV1 and TRPV4 activation reduces microglial activation and reduces subsequent neuronal death), others are involved in more pathological outcomes (e.g., TRMP2 activation causes microglial nitric oxide production, leading to central sensitisation and neuropathic pain), although these outcomes may depend on the physiological context [48]. Moreover, the downstream consequences of TRP channel activation in other cells (e.g., astrocytes, neurons) may affect microglial function. For example, B1 receptor activation in sensory neurons, induced by des-Arginine9-bradykinin (DABK) in mouse spinal cord, activates TRPA1, which subsequently causes microglial activation [54].

Interestingly, the mechanosensitive Piezo1 (Piezo type mechanosensitive ion channel component 1) channel protein is most highly expressed in microglia and endothelial cells within the mammalian brain [55] and has been implicated in microglial function. Inhibiting Piezo1 pharmacologically or genetically protects BV-2 microglia from acute hyperglycaemia damage, in vitro [56]. Piezo1 mRNA is aberrantly downregulated in neurons and upregulated in amyloid-reactive astrocytes that surround amyloid plaques, in post-mortem AD brains and in an aged rat model of AD (TgF344-AD) [47,57]; additionally, it is upregulated in the aging rat brain [47]. It may be the case that Piezo1 is aberrantly regulated in microglia in AD as well, leading to altered mechanotransduction, or it may be hyperactivated due to the increased stiffness of aged or amyloid-plaque-rich neural tissue. There is also evidence that Piezo1 channel activation may regulate the Hippo signalling pathway [58], another critical mechanosensitive pathway.

## 4. The Hippo Signalling Pathway

In addition to TRP and Piezo1 mechanosensing pathways, another extensively studied mechanosensing pathway is the Hippo signalling pathway, which has generated recent interest in preventing neurodegeneration and potentially encouraging neuroregeneration. The Hippo signalling pathway has been implicated classically in growth control, but it is a multi-purpose pathway that has been recently suggested as a therapeutic target in the prevention of neurodegeneration due to its function in the CNS [59]. The canonical Hippo pathway was initially discovered in the fruit fly *Drosophila melanogaster* and was observed to play a key role in the regulation of cellular proliferation and apoptosis [60,61]. This pathway is evolutionarily conserved in mammals and comprises a protein kinase cascade that regulates the downstream effector Yes-associated protein (YAP). The Hippo pathway plays an important role in the regulation of many diverse cellular functions, including the mechanical sensing of cellular structure, survival, tissue regeneration and organ homeostasis [62,63,64,65].

**Figure 2:**
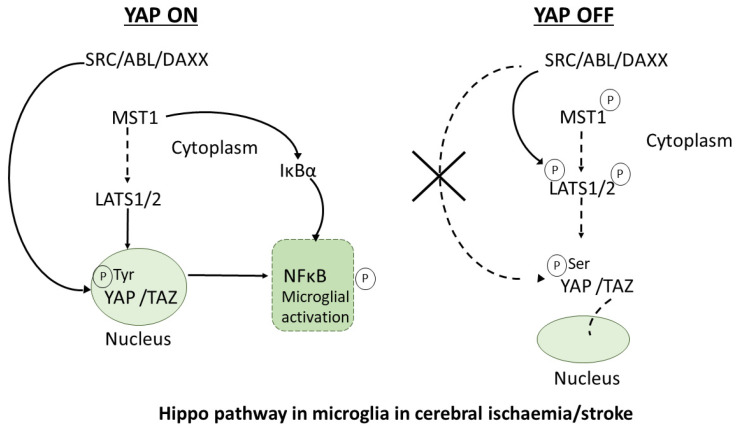
Hippo pathway components that are known to function in microglia under cerebral ischaemia or stroke conditions (see Section 4.2). On YAP activation by phosphorylation at tyrosine (Tyr) residues (‘YAP ON’), YAP translocates to the nucleus to initiate specific gene transcription. On YAP inactivation (phosphorylation at serine (Ser) residues), YAP translocates to the cytoplasm (‘YAP OFF’). Phosphorylation (P) at the relevant residues by the upstream kinase is indicated by arrowed lines. Solid lines indicate experimentally proven pathways. Dotted lines indicate potential or likely pathways that have not yet been shown experimentally. Components in the nuclear and cytoplasmic components are labelled.

In mammals, the core components of the Hippo pathway (summarised in Figure 2) comprise two main kinase families—mammalian sterile 20-like protein kinase 1/2 (MST1/2) and large tumour suppressor 1/2 (LATS1/2). The other key components of this pathway include the downstream transcriptional effector proteins, YAP, and transcriptional coactivator with PDZ-binding motif (TAZ), as well as several adaptor proteins that stabilise the core kinases, including Salvador homolog 1 (SAV1) and MOB1 (MOB kinase activator 1 A/B). The downstream effector YAP plays a key role in both cellular sensing as well as growth control, and can be negatively controlled via phosphorylation by LATS1 and LATS2 and positively controlled by activation by the extracellular matrix [63,66,67,68]. When YAP is active, it translocates into the nucleus to bind to a key transcriptional factor known as TEAD (TEA domain containing transcription factor) in order to enhance cell proliferation, differentiation and survival, and when YAP is phosphorylated by LATS1/2, it becomes inactive and translocates into the cytoplasm, where it is sequestered by 14-3-3 proteins [54].

### 4.1. Activation of the Hippo Pathway by Mechanotransduction

The Hippo signalling pathway effector Yes-associated protein (YAP) has been shown to act as a ‘tension sensor’ (sensing the mechanical force of tension) in various cancer cell lines. Plating cells on surfaces of varying stiffness affects not only cellular shape, but also the activation state of YAP [69,70]. An axis of tension can either inactivate YAP by the canonical Hippo signalling pathway (see Section 4) or activate YAP by the action of the ECM and its constituent components [71]. For example, this is clearly observable in colon adenocarcinoma cells, which form a columnar shape when fully differentiated and form an apical (uppermost part of the cell) and a basolateral (side and bottom of the cell) domain, allowing for the segregation of proteins to the correct cellular compartments [72]. In well-differentiated cells, the apical domain allows for the correct localisation of the canonical Hippo signalling pathway proteins MST and LATs to their correct locations for subsequent activation, and thus they can phosphorylate and inactivate YAP [72]. Conversely, if cells lack a clearly defined apical domain (e.g., in the case of skin keratinocytes), a basal ECM signal can override any apical signal and subsequently promote the activation of YAP instead, via an integrin–Src axis (where integrins provide cells with connection to the basement substratum and extracellular signals) [72].

Recently published studies have elucidated the importance of the Hippo signalling pathway in the nervous system, as the Hippo signalling pathway has been established in neuroinflammation, neuronal cell differentiation and neuronal death [59,73,74]. As mentioned above, the CNS can undergo mechanotransduction during initial neuronal cell development, and this commonality with the Hippo signalling pathway, which has been shown to use YAP as a tension sensor in the context of mechanotransduction and polarity [69,72], makes it an obvious target to explore any links [57,58]. The expression levels of the Hippo signalling pathway core components have been examined in the CNS, and YAP as well as the core kinases MST1/2 were found to be highly expressed in a wide variety of cell types within the brain, including microglia cells and during neuronal development [1,55,75,76]. However, the role of the Hippo pathway in neurodegenerative disease has not been well described, and not much is known about the role of YAP in the CNS. However, it is already known that in microglia, genetic depletion of the extracellular matrix (ECM) protein FERM domain-containing protein kindlin3 (K3) results in defective membrane tension, with a strong effect on YAP activation in the nucleus [8]. With regard to microglia, resting (homeostatic) microglial cells are ramified and extremely sensitive [77,78], and could be thought of as tension/mechano-sensors in the way that they can react to external mechanical stimuli [78]. As this is similar in action to the role that YAP plays in other cell lines, we hypothesise that YAP and the Hippo pathway may act as a tension sensor in microglia to allow for the responsive behaviour of microglia.

In the context of mechanotransduction, regulation of the actomyosin cytoskeleton is a key part of cellular dynamics. Remodelling of the ECM and cell migration requires the actomyosin cytoskeleton [79]. The Rho GTPase Rho and its downstream effector Rho Kinase (ROCK) are involved in the phosphorylation of the myosin light chain to promote contractility of the ECM and matrix stiffening [80]. This results in the activation of Src, which subsequently activates YAP and thus further activates myosin in a positive feedback loop. In microglia, the activation of ROCK results in changes in microglial phenotype: the increase in ROCK activity activates various mechanisms of inflammation and is associated with an increase in motility, upregulation of reactive oxygen species (ROS) and a subsequent release of inflammatory cytokines [81]. This phenomenon in microglia could potentially be modulated by YAP.

The other key Rho GTPase, which is important for mechanotransduction, is the small GTPase CDC42. It has been shown previously that, if CDC42 is depleted in cells, polarity is lost and YAP becomes active [72]; this is due to the cells losing their structure, becoming more flat and thus mimicking a more migratory type of cell. It was shown in post-mortem brain samples that Cdc42 expression was upregulated in AD patients; thus, this potential loss of polarity could contribute to YAP activation and thus increase pro-inflammatory cytokines, contributing to the progression of AD [82]. It has also been shown that a novel inhibitor of ROCK could actually decrease AD disease severity when used and could potentially have some crosstalk with the Hippo signalling pathway, but this is yet to be tested experimentally [83].

### 4.2. Evidence for Hippo Signalling Pathway Function in Microglia, from Cerebral Ischaemia and Nerve Injury Studies

In addition to mechanosensation, microglia can change into different functional states (from homeostatic to differently activated states) and thus vary levels of proliferation as well as undergo apoptosis, key traits which are also regulated by the Hippo signalling pathway [84]. MST1, a serine-threonine kinase, has well-known pivotal roles in organising immune cell function, as MST1 null mutation in humans causes primary immunodeficiency, caused by a loss of naïve T-cells [85], and MST1-deficient mice show defects in T-cell function resulting in impaired long-term humoral immunity [86]. Recent studies on molecular signalling have also revealed the participation of the Hippo/MST1 signalling pathway in the activation of microglia cells in stroke patients, and suppression of MST1 expression was found to rescue neuronal apoptosis in a wide range of injury-induced neurodegenerative disorders, such as stroke and haemorrhage. This mechanism could be potentially harnessed in treating such neurodegenerative pathologies in the future by preventing further neural damage [59].

Microglial activation is known to be an essential step for oxidative stress-induced neuronal cell death in ischemic stroke-induced immune alteration [75,87], and MST1 signalling is crucial to inducing this process [75,87]. Knockout of MST1 in microglia protects against acute ischaemia/reperfusion (I/R)-induced neuroinflammation and brain injury [88]. In the I/R condition, MST1 directly phosphorylates residues S32 and S36 of the substrate protein nuclear factor of kappa light polypeptide gene enhancer in B-cell inhibitor alpha (IκBα) (Figure 2); therefore, it regulates the activation of nuclear-factor-kappa B (NF-κB) signalling in microglia [88]. The inhibition of MST1 in microglia suppressed NF-κB signalling and microglial activation (Figure 2), showing that MST1 has a protective function in microglia-induced neuroinflammation. MST1 signalling and kinase activity is activated in this microglial context by upstream key signalling molecules such as Src, c-Abl and Daxx [23,75] (Figure 2). Src kinase was found to directly phosphorylate the tyrosine 433 site of MST1, leading to MST1 activation and thus regulating MST1–IκBα signalling throughout microglial activation [23,75] (Figure 2). Hence, the Src–MST1–IκBα signalling axis is a significant player in neuronal death via microglial activation (59). MST1 regulation of the ischemic response seems to be via activation of the protein FOXO and is Hippo independent, but it is important not to rule out that MST1 might also be acting directly upon YAP/TAZ in microglia (Figure 2); activity changes in both YAP and TAZ have also been shown during the I/R condition in mice and are associated with worse neurological outcomes [89].

It is also possible that Src also activates YAP in microglia (Figure 2), independently of its activation of MST1 (Figure 2); however, this has not been shown directly in microglia and is based on observations found in other model systems and needs to be experimentally tested. This could again suggest that mechanotransduction could be playing a key role in microglia, due to the involvement of extracellular matrix membrane receptors. For example, integrins can subsequently send a signal once mechanotransduction occurs outside the cell in order to activate SRC in various tissues [66,90]. The stiffness of the ischaemic brain microenvironment has not been well studied, but a recent study suggests that ipsilateral (same hemisphere) brain tissue stiffens directly after ischaemia in mice [91]; it may be possible that microglial SRC senses this increased stiffness and acts via microglial MST1 and/or YAP to coordinate the microglial inflammatory response in this condition. Nevertheless, the role of activated microglia in an ischaemic environment and the role of inflammatory mediators in this process still require much research.

Reports related to peripheral nerve injury also support a role for Hippo signalling in microglia, where a predominance of TAZ as opposed to YAP expression in microglia from the spinal cord dorsal horn of rats suggests that TAZ may have a proliferative role in response to nerve injury. This could be independent to the canonical Hippo signalling pathway, as it has been shown that TAZ can be phosphorylated independent of LATS signalling [92,93].

### 4.3. The Hippo Pathway in Microglia and Links to AD Pathogenesis

As microglia are now highly implicated in AD pathogenesis, it is pertinent to hypothesise that molecular pathways in microglia may be implicated or altered in AD pathogenesis and disease. Interestingly, a recent proteomic study showed reduced expression of Hippo pathway components across all sampled regions of the diseased post-mortem human AD brain [94], potentially supporting the hypothesis that the Hippo pathway may be dysregulated in severe neurological disease processes.

Microglia in the healthy human brain express high levels of the Hippo pathway components TAZ/MST/SAV1/LATS but have a relatively low expression of YAP [76]. Additionally, YAP1 has been shown to be expressed in isolated microglia from embryonic mouse brain by RNA-Seq but decreases in expression in microglia from adult mouse brain. YAP1 is also expressed more highly in fusiform gyrus and temporal cortex from patients with Alzheimer’s disease, compared to controls [95]. With regard to AD pathogenesis, Hippo/YAP signalling has been shown to affect the production of pro-inflammatory cytokines associated with microglial activation, which are induced by Aβ, in a microglial cell line [96]. Aβ reduced the expression of YAP in microglia, and the YAP agonist XMU-MP-1 or YAP overexpression in microglia resulted in a decrease in proinflammatory cytokine expression (Figure 2) by affecting levels of glial-cell-line-derived neurotrophic factor (GDNF) [96]. On the other hand, the YAP antagonist verteporfin or YAP knockdown had the opposite effect [96]. This suggests that YAP expression in microglia is associated with a reduced proinflammatory (potentially harmful) microglial activation state.

YAP has also been found to be an important upstream regulatory ‘hub’ gene in the initiation of AD by transcriptional analysis of AD brains, although whether this was in microglia, neurons or other cells was not analysed [97]. YAP was downregulated in hippocampus tissue from a transgenic mouse model of AD pathology at early stages (2 months before Aβ or tau pathology) and in hippocampi from incipient AD patients [97], suggesting that YAP changes are present in early disease stages. Interestingly, YAP1 was found to be significantly correlated as an AD-related disease gene in the cell rich grey matter [98]. Knockdown of YAP resulted in an increase in Aβ1-42 and tau phosphorylation, and in changes in proteins associated with Aβ production (BACE1, PSEN1, PSEN2, and Nicastrin) and tau phosphorylation (CDK5, GSK3α/β) [97]. Additionally, neuronal cell death in the initial stages of AD was also found to be a Hippo signalling pathway-dependent cell necrosis [99]. Another key finding was that intracellular Aβ aggregates restricted YAP from entering the nucleus, thus causing a decrease in nuclear YAP levels in the cortical neurons of AD and mild cognitive impaired patients (MCI). Further analysis of this found that immunoprecipitation of cerebral cortex tissues from AD patients found a clear interaction between YAP and Aβ, further strengthening the link between the Hippo pathway and AD [99].

YAP signalling has also been recently identified as a critical regulator of a novel type of cell necrosis, TEAD/YAP-transcription-dependent-necrosis (TRIAD), in the neurodegenerative disorder Huntington’s disease [100,101]. It is therefore very possible that YAP also regulates TRIAD in AD. Taken together, these findings suggest that aberrant downregulation of YAP may have a role in early AD pathogenesis; it can be hypothesised that this could be perhaps by reducing healthy clearance of Aβ or tau by microglia, and/or by permitting proinflammatory microglial activation. As these findings do not specifically focus on microglial YAP, it will be important to identify the CNS cells contributing to this process.

Conversely, YAP was upregulated in CNS tissue from old AD mice and severe AD patients [97,98]. It would be interesting to determine if this represents compensatory expression changes or secondary cellular changes due to extensive neurodegeneration at these stages, or if cell death is directly regulated by YAP at these later stages as well as at initial stages.

In contrast to low expression in microglia, YAP is highly expressed in astrocytes [94,97]. Astrocytes are the predominant CNS glial cell, which interact with microglia and have an important role in the activation of immunological pathways, including in AD [102]. Indeed, deletion of YAP in an animal model shows that astrocytes are able to stimulate microglial activation and cause blood–brain dysfunction, possibly through the secretion of cytokines and chemokines [90,103]. In this way, the Hippo pathway could also function on microglia as a secondary cell effector. It would be useful to examine this experimentally in AD co-culture or in vivo models.

One factor that is crucial in any immune response and activation is an association with cellular metabolism [104]. During the activation of microglia, cellular metabolism is reprogrammed to enable activation and phagocytosis [105], with glucose being a major energy source [106]. Exposure to amyloid beta (Aβ) has also been shown to induce metabolic switching in microglia, resulting in diminished immune responses that are dependent on glycolytic reprogramming [107]. The chemokine CX3CL1, which is highly associated with AD, has also been shown to inhibit microglial activation through the induction of a metabolic switch. This occurs by changing the phenotype of the microglia by reducing the expression of genes related to the glycolytic pathway and increasing the expression of genes related to the oxidative pathway, restraining inflammation [108].

It is now recognised that cellular metabolism can be regulated by the Hippo pathway [109]. Modulators of these processes of regulation are not fully understood but may include transcription factors, receptors, ion channels and processes such as oxidative stress, hypoxia and metabolic cues from the substrates themselves [110]. To this effect, AMP-activated protein kinase (AMPK), a master regulator of cellular energy homeostasis, has also been linked to AD brain pathology through its regulation of protein synthesis and autophagy [111]. AMPK can phosphorylate YAP and therefore suppress its activity following periods of energy stress in various cancer-derived cell lines [112].

AMPK can in theory also regulate YAP via mechanotransduction. If force is applied to cell–cell contacts, this can cause the protein Liver Kinase B1 (LKB1) to activate AMPK. LKB1 recruits AMPK to the cell contact protein E-cadherin and forms a mechanotransduction complex, thereby resulting in actomyosin contractility [113]. LKB1 itself has been shown to regulate YAP activation [114], thus potentially resulting in a mechanotransduction-induced complex in the CNS. This finding may provide additional promising therapeutic targets (e.g., AMPK) for the treatment of AD.

Another potential area to focus upon is the purinergic receptor family. This receptor family is involved in neuroinflammation and may regulate functions of various neuronal cell types, including microglia. It was shown that the P2Y2R-mediated activation of YAP controls human cardiac progenitor cell migration and activation while inactivating Lats1 [115]. Another potential link is that Src is involved in the activation of purinergic receptors in microglia [116]; therefore, it would be interesting to assess this link further in microglia.

## 5. Conclusions

Microglia can sense and respond to their physical microenvironment, but the molecular mechanosensing pathways in microglia are not yet well elucidated. Components of the Hippo pathway are expressed in microglia, are altered in CNS injury or inflammation, and have potential, although not fully characterised, links to AD pathologies. Specifically, microglial YAP and MST1 appear to be putative regulators of microglial biology in the context of Alzheimer’s disease pathology. As well as further studies detailed previously, additional studies would be useful to address the putative role of Hippo/YAP signalling in microglia and in AD. It would be useful to define expression at the mRNA and protein level of specific Hippo components, both in isolated microglia from control and AD patient brains, and in microglial cell models; in particular, studying this in hiPSC-derived microglia from control and AD patients with known mutations (e.g., APP) would allow for temporal analysis of Hippo/YAP component expression and correlation with cellular changes or pathology. Activation or inhibition of the Hippo pathway (e.g., by pharmacological activation or CRISPR knockout) in human-derived microglial cell lines, or in control hiPSC-derived microglia, in co-culture with neurons, and measuring the resulting effect on microglial activation, Abeta/tau production, phosphorylation, and aggregation, as well as neuronal death, would be useful in defining any specific roles for microglial Hippo/YAP in some of the known pathogenic processes leading to AD. These studies may also suggest whether Hippo/YAP signalling could represent a promising therapeutic target for AD, perhaps particularly in the early stages of the disease. Additionally, it would be interesting to examine if Hippo/YAP signalling has a role in other neurodegenerative diseases or injury with neuroinflammatory components, such as multiple sclerosis, Parkinson’s disease, and traumatic brain injury.

Hippo pathway components are increasingly becoming targets for diseases such as cancer as well as regenerative medicine. Chemical inhibitors have been identified to target the Hippo signalling pathway. Tumorigenesis in different cancer cell lines has been suppressed by small molecule inhibitors that target the downstream components of the Hippo pathway [117]. These chemical inhibitors can be tested in neurodegenerative disorders for their efficiency in their potential of delaying the onset and progression of the disease. Much more research is needed into the Hippo pathway and its activators in microglia mechanotransduction, particularly in AD and other neurodegenerative diseases; potential activators could be tissue stiffness (including amyloid plaques), astrocytes, metabolic changes, or other factors. In several neurodegenerative diseases, the Hippo signalling pathway is hyperactivated; therefore, it is feasible that chemical inhibitors modulating Hippo pathway components could be a therapeutic target for blocking neurodegeneration by manipulating the Hippo pathway through core kinases such as MST1/2 or by the effector YAP and its interaction with TEAD [118].
